# High‐Density RNA Microarrays Synthesized In Situ by Photolithography

**DOI:** 10.1002/anie.201806895

**Published:** 2018-10-19

**Authors:** Jory Lietard, Dominik Ameur, Masad J. Damha, Mark M. Somoza

**Affiliations:** ^1^ Institute of Inorganic Chemistry Faculty of Chemistry University of Vienna Althanstraße 14, UZA II 1090 Vienna Austria; ^2^ Department of Chemistry McGill University 801 Rue Sherbrooke O Montréal QC H3A 0B8 Canada

**Keywords:** microarrays, phosphoramidite chemistry, photolithography, RNA, RNase HII

## Abstract

While high‐density DNA microarrays have been available for over three decades, the synthesis of equivalent RNA microarrays has proven intractable until now. Herein we describe the first in situ synthesis of mixed‐based, high‐density RNA microarrays using photolithography and light‐sensitive RNA phosphoramidites. With coupling efficiencies comparable to those of DNA monomers, RNA oligonucleotides at least 30 nucleotides long can now efficiently be prepared using modified phosphoramidite chemistry. A two‐step deprotection route unmasks the phosphodiester, the exocyclic amines and the 2′ hydroxyl. Hybridization and enzymatic assays validate the quality and the identity of the surface‐bound RNA. We show that high‐density is feasible by synthesizing a complex RNA permutation library with 262144 unique sequences. We also introduce DNA/RNA chimeric microarrays and explore their applications by mapping the sequence specificity of RNase HII.

High‐density DNA microarrays refer to extensive libraries of oligonucleotide sequences immobilized onto a surface.^1^ Photolithography and inkjet printing are the two principal approaches that can accomplish high‐density in array fabrication. Both methods adopt the cycle‐based phosphoramidite chemistry,^2^ albeit with minor changes, resulting in efficient large scale in situ oligonucleotide synthesis.^3^ The historical spectrum of DNA microarray applications has progressively expanded to include genotyping,^4^ gene expression,^5^ gene synthesis,^6^ protein binding site identification,^7^ to mention but a few.^8^ Displaying an entire library on defined spots (“features”) and having the ability to address each sequence combination individually makes microarrays particularly well suited to the analysis of DNA binding motifs. Given the structural and functional diversity of ribonucleic acids, it is equally appealing to be able to offer RNA microarrays as platforms to better understand RNA chemistry and biology.

The development of RNA microarrays was limited for a long time to the spotting of pre‐synthesized RNA strands.^9^ Meanwhile, the fabrication methods for DNA arrays have improved and now support the preparation of longer oligonucleotides, in higher quality and at lower costs.^5^, ^10^ For those reasons, “on‐chip” DNA synthesis serves as an ideal template for RNA polymerization. The enzymatic transcription of immobilized DNA into RNA has been reported,^11^ and recently extended to DNA arrays synthesized by photolithography.^12^ While this elegant method capitalizes on the robustness of DNA phosphoramidite chemistry, the combined DNA synthesis and enzymatic processing adds to the total complexity and production time. In addition, the chemical space that can be explored with enzymatic fabrication is limited to four nucleotides.

Perhaps the direct synthesis of oligoribonucleotides from RNA phosphoramidites is the most instinctive approach to the fabrication of RNA microarrays. Transitioning between DNA and RNA synthesis on the solid‐phase only requires a change of monomers. However, this simple procedure does not carry over to microarray synthesis. Indeed, in situ synthesis prohibits the use of 2′‐*O*‐silyl protected nucleosides,^13^ since a fluoride treatment for 2′‐OH deprotection degrades the glass substrate. A new set of 2′‐protected RNA synthons therefore needed to be developed to undertake in situ array fabrication. We already reported on the preparation of RNA phosphoramidites protected at the 2′‐OH position with an acetal levulinyl ester (ALE).^14^ Using hydrazine, the levulinyl ester can be cleaved under mild conditions, while the acetal moiety offers the additional synthetic advantage of preventing 2′ to 3′ migration of acyl groups. This levulinyl‐based protection strategy allowed for the complete “on‐support” deprotection of RNA oligonucleotides,^15^ a concept which naturally resembles microarray synthesis. To be fully compatible with photolithography, a 5′ photosensitive nitrophenylpropoxycarbonyl (NPPOC)^16^, ^17^ group was installed on 2′‐*O*‐ALE ribonucleosides, which were then transformed into their corresponding phosphoramidites (Figure 1).

**Figure 1:**
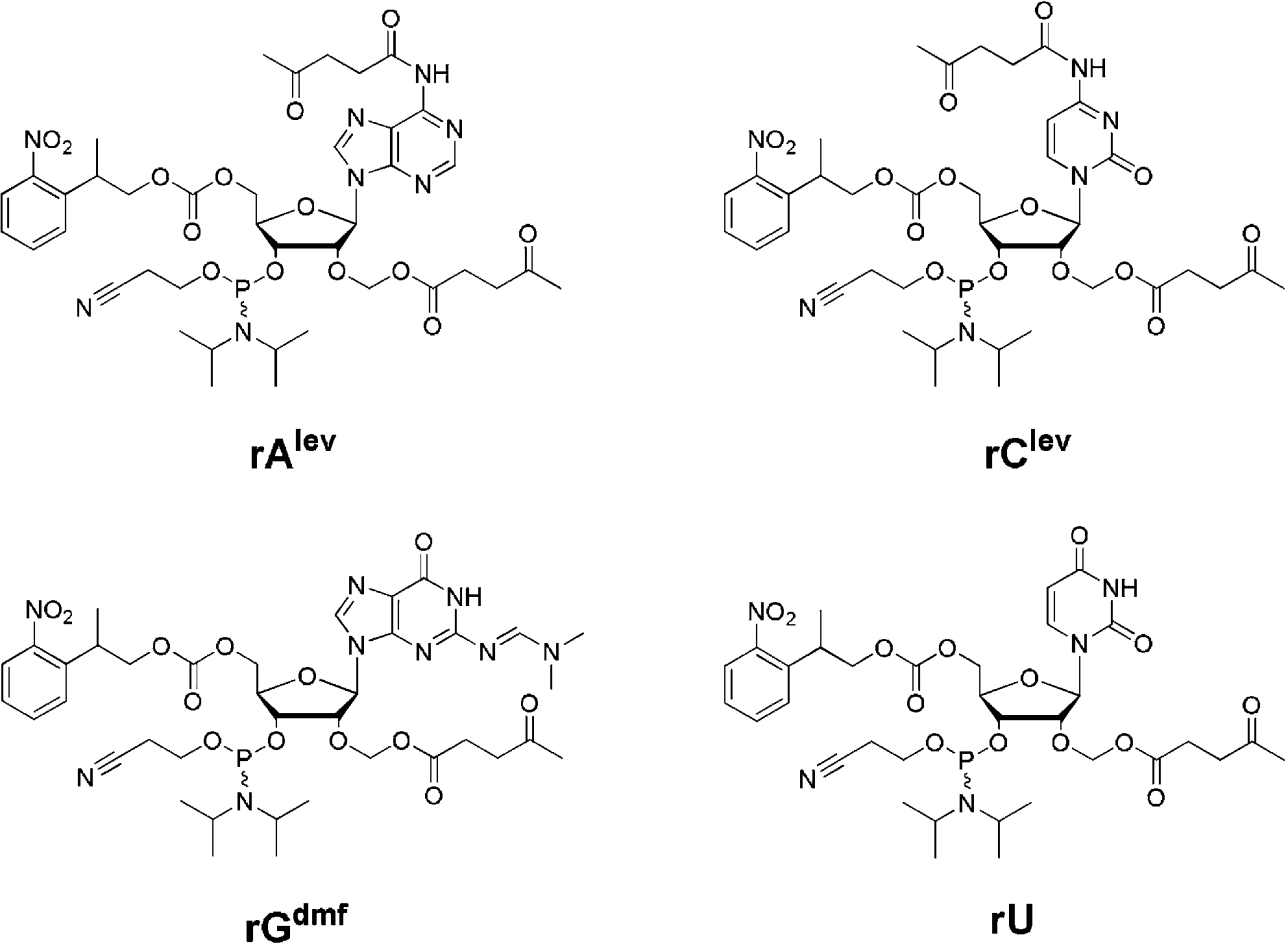
Chemical structures of the 5′‐NPPOC 2′‐*O*‐ALE RNA 3′‐phosphoramidites used for in situ microarray synthesis by photolithography. lev=levulinyl, dmf=dimethylformamidine.

Preliminary tests of in situ RNA array synthesis with those novel monomers showed good‐to‐average coupling efficiencies, and short homopolymers of rU and rA were found to correctly hybridize to their complements. An RNase A assay on sequences containing a single rU incorporation served as an additional proof‐of‐concept.^18^ We have extended our method to the incorporation of all four bases and now wish to describe the synthesis of mixed‐base, high‐density RNA microarrays by in situ photolithography and their potential as nucleic acids libraries in the study of RNA‐ligand interactions.

We first revisited the coupling efficiencies of all RNA phosphoramidites, which can now be obtained in gram quantities and at high purity. A series of homopolymers of each base and of varying lengths, representing up to 12 consecutive couplings of the same base, were synthesized and labelled at the 5′ end with a Cy3 amidite (Figure S1 in the Supporting Information). The arrays were scanned immediately after synthesis. The stepwise coupling yields were found to range between 99 % (rG, rC) and 99.9 % (rU, rA), similar to the best results typically obtained in DNA synthesis (Table 1 and Figure S2).

**Table 1:** Stepwise coupling efficiency of 2′‐*O*‐ALE RNA phosphoramidites

Parameter	rA	rC	rG	rU
Coupling time (min)	5	5	5	2
Coupling efficiency (%)	>99.9	99.3	99.1	>99.9

We then went on to synthesize, on one surface, the DNA and RNA forms of a 25mer sequence containing all four bases (GTC ATC ATC ATG AAC CAC CCT GGT C). After synthesis, the microarrays are deprotected in a stepwise manner. First, the cyanoethyl protecting groups of the phosphodiester bonds are removed in Et_3_N/ACN 2:3 for 90 min, then the nucleobases and 2′ hydroxyl functions are simultaneously removed in a buffered solution of 0.5 m hydrazine hydrate in pyridine/AcOH 3:2 for 2 h. We however found that DNA sequences cannot be completely deprotected via hydrazinolysis only and require an extra ethylenediamine (EDA) step. Next, the oligonucleotides were hybridized to their Cy3‐labelled DNA complement and showed similar hybridization intensities (Figure 2) for the DNA:DNA‐Cy3 and RNA:DNA‐Cy3 duplexes. This is, to our knowledge, is the first instance of a four‐base RNA array synthesized in situ.

**Figure 2:**
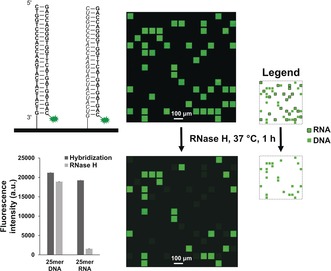
Schematic representation of DNA (bold) and RNA (italic) sequences hybridized to the Cy3‐labelled DNA complement. A small scan excerpt (ca. 5 % total synthesis area) of the hybridized array is shown to the right. Dark gaps between features only contain a linker (T_10_). Spot size is approximately 70×70 μm. The same array is scanned after treatment with RNase H (5 U) for 1 h at 37 °C. The legend on the right identifies the RNA and DNA features in the scanned array. The fluorescence intensities (arbitrary units) are then plotted before and after RNase H treatment. Error bars are standard error of the mean (SEM).

To confirm the identity of the 25mer DNA and RNA oligonucleotides in Figure 2, an RNase H assay was performed on the hybridized chip. After 1 h at 37 °C in presence of RNase H, the fluorescence of the RNA:DNA‐Cy3 duplex was dramatically reduced, while that of the DNA:DNA‐Cy3 remained, as expected, unchanged. Attempts at rehybridizing the microarray to the same complement at the same temperature did not restore fluorescence signals for the RNA:DNA duplex, demonstrating significant selective cleavage of the RNA strand.

Finally, not only can DNA and RNA synthesis be carried out in parallel, RNA phosphoramidites can also be incorporated within a DNA oligonucleotide. For instance, the above‐mentioned DNA 25mer substituted with rU units at every dT position (six incorporations) correctly hybridized (Figure S4). The slightly weaker fluorescence signals for the rU‐modified 25mer relative to the pure DNA sequence may be attributed to multiple A‐ and B‐form helical junctions within the DNA/RNA duplex.

Maskless array synthesis (MAS) relies on a digital micromirror device (DMD) in an imaging system to pattern and deliver 365 nm light onto spatially defined, micrometer‐sized features on the glass substrate.3a, 19 The layout of the microarray is controlled by a computer, which instructs the DMD to tilt the necessary mirrors for reflection of the UV light onto the surface, triggering photodeprotection only on the receiving features (Figure 3 A). Our MAS setup is equipped with a DMD of 1024×768 mirrors, resulting in a maximal achievable density of 786 432 features per array.^19^, ^20^ The number of sequence combinations of nine nucleotides can be expressed as 4^9^ (262 144 sequences) and fit comfortably on a single array, along with replicates (Table S1). We envisaged the fabrication of a high‐density RNA microarray hosting the entire permutation library of a 9‐nt sequence (Figure 3 B). The library of 9‐nt sequence permutations, flanked by fixed 5′ and 3′ tails producing a single‐stranded 28mer RNA was synthesized by photolithography, deprotected and hybridized to a 28‐nt DNA strand (Figure 3 C, Figures S5 and S6). As expected, the full‐match sequence gave some of the brightest fluorescence signals, outranked only by a handful of single‐mutated sequences. This outcome may be the result of smaller sample size (2 replicates for the mutated sequences vs. 7000 replicates for the match). Still, the hybridization signals of 97 % of all sequence permutations were contained within the lowest quartile of recorded fluorescence, as was observed for the DNA version of the library (Tables S2 and S3), suggesting comparable sequence discrimination during hybridization in DNA and RNA arrays.

**Figure 3:**
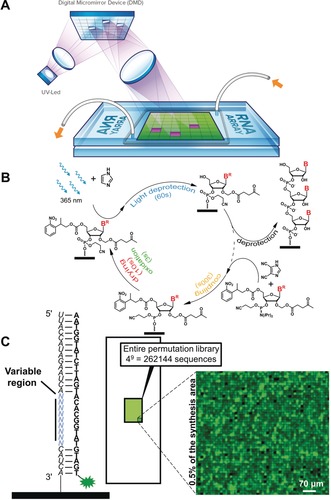
A) Schematic representation of the principle of microarray synthesis by photolithography using MAS. UV light (from a UV‐LED source) reflected on the tilted micromirrors in the DMD is projected onto the surfaces of two stacked glass slides and triggers the removal of the photosensitive NPPOC protecting group only on the features colored in purple. Features colored in green do not receive UV light during this exposure event. White tubes and orange arrows indicate the flow of solvents and reagents between the slides. B) Schematic representation of the phosphoramidite‐based coupling cycle used to grow RNA oligonucleotides in situ. C) Right: excerpt (<0.5 % of total synthesis area) of an RNA microarray scan after hybridization of the 9‐nt RNA permutation library to a Cy3‐labelled DNA strand (left). Spot size is 14×14 μm.

To highlight the potential applications of complex arrays of RNA and mixed DNA and RNA chemistries in molecular biology and chemical biology research, we chose RNase HII, an enzyme whose preferred substrates are double‐stranded DNA carrying a single RNA base^21^ and questioned whether RNase HII displays any sequence preference. Microarrays are well‐suited to perform enzyme/ligand‐binding experiments and are an established alternative to more standard methods.^22^ We thus designed and synthesized a library of DNA hairpins containing a single RNA base. The hairpin consists of a 9‐nt stem, a 4‐nt loop and is terminated with a Cy3 dye at the 5′‐end (Figure 4). Within the stem, we selected a variable region of 5 consecutive nucleotides, the middle position being the RNA nucleotide. All possible permutations (4^5^=1024 sequences) in the variable region were synthesized in multiple replicates, deprotected and subjected to RNase HII‐mediated cleavage. A final treatment with water at 40 °C ensured complete removal of the cleaved portion of the hairpin. RNase HII‐mediated cleavage results in loss of fluorescence, as treating the array in buffer alone led to no significant difference in fluorescence relative to the DNA‐only hairpin. Interestingly, we found that the hairpin sequences were not all cleaved to the same extent, but with noticeable disparities spanning a 40 % range. The least‐cleaved nucleotide combination, TTrGCT, only lost 30 % of its initial fluorescence relative to the DNA hairpin, while the most‐cleaved sequence, GCrCCC, decreased by 70 %. Notably, the DNA hairpin containing a single rU insert within the TCCT loop was also found to be cleaved by up to 20 %, indicating some level of structural recognition by RNase HII even on unpaired ribonucleotides, which is not unheard of.^23^

**Figure 4:**
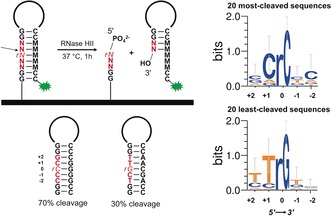
Left: Schematic representation of the DNA hairpin array design containing a single RNA insert (in italic) and the chemical outcome of enzymatic cleavage mediated by RNase HII. In red is the 5‐nt long variable region. The cleavage site is represented by an arrow. M stands for the complementary nucleotide to the dN or rN nucleotides. Hairpin sequences below represent the best and worst substrates for RNase HII‐mediated cleavage. Right: Sequence motifs assembled from the collection of the top 20 most‐ and least‐cleaved hairpin sequences.

The vast majority of sequences (846 out of 1024) showed cleavage rates between 40 and 60 %, and the remaining combinations displayed lower (30–40 %) or higher (60–70 %) cleavage rates. We then searched for sequence motifs within the low and high cleavage subgroups. The large majority of highly‐cleaved hairpins (100 sequences) contains rC as the RNA base, and rU in a few select cases. In fact, rC becomes the only RNA base found in the 20 most‐cleaved hairpins (Figures 4 and S7). Conversely, poorly cleaved substrates are largely populated with rG as the RNA nucleobase, and rA in a few select cases. Again, the 20 least‐cleaved sequences always contain rG. Of importance is the identity of the DNA nucleobase found 5′ to the RNA insert (position +1), particularly since the 5′‐DNA‐RNA‐3′ junction is the scissile phosphodiester bond.^24^ We found that cytosine is the preferred base in the best RNase HII substrates, while thymine is very frequently found 5′ to the RNA in the worst substrates. Further discussion of the results can be found in the Supporting Information.

In summary, we have presented the direct fabrication route for the first in situ‐synthesized RNA microarrays that does not require DNA transcription. The photolithography approach allows us to explore large combinatorial libraries and to reach the same high‐density and sensitivity as in situ synthesized DNA arrays. Enzymatic assays confirm RNA identity and demonstrate the usefulness of the method. The in situ RNA array synthesis method not only supports the mixing of DNA and RNA chemistries to produce DNA/RNA chimeric microchips, which we described herein, but also paves the way for the flexible incorporation of both non‐canonical and non‐natural nucleotides.22c, 25

## Conflict of interest

The authors declare no conflict of interest.

## Supporting information

As a service to our authors and readers, this journal provides supporting information supplied by the authors. Such materials are peer reviewed and may be re‐organized for online delivery, but are not copy‐edited or typeset. Technical support issues arising from supporting information (other than missing files) should be addressed to the authors.

Supplementary
