# Supplementary material for: High‐Density RNA Microarrays Synthesized In Situ by Photolithography
Source: Angew Chem Int Ed Engl. 2018 Oct 19;57(46):15257–61. doi: 10.1002/anie.201806895 (PMC6237118; doi:10.1002/anie.201806895)
Supplement: Supplementary file 1 — Supplementary [file ANIE-57-15257-s001.pdf]

Angewandte Chemie
Eine Zeitschrift der Gesellschaft Deutscher Chemiker

Supporting Information

High-Density RNA Microarrays Synthesized In Situ by Photolithography

Jory Lietard, Dominik Ameur, Masad J. Damha,* and Mark M. Somoza**

anie_201806895_sm_miscellaneous_information.pdf

Supporting Information

Table of Contents

General Experimental Methods **2**

General Microarray Fabrication Procedure **2**

Slide functionalization 2

Nucleic acid synthesis by photolithography 2

Synthesis area, number of features and density 3

Deprotection, Hybridization and RNase H assays **4**

DNA microarrays 4

RNA microarrays 4

Hybridization 4

RNase H assay 6

Determination of the coupling efficiency **7**

Deprotection of Cy3-labelled RNA arrays and RNA degradation 9

Construction of the 4^9 high-density RNA library **12**

RNase HII assays **17**

Further discussion on the results of the RNase HII assay 20

- *Identity of the DNA nucleobases around the RNA insert* 20
- *Number of GC versus AT base pairs in the hairpin stem* 21

References **21**

General Experimental Methods

All solvents were purchased in anhydrous form from Biosolve or Sigma Aldrich and stored under activated 4 Å molecular sieves. Chemicals and reagents for microarray synthesis were purchased from Sigma Aldrich and ChemGenes and used with further purification. DNA phosphoramidites were obtained from Sigma Aldrich, Orgentis or Flexgen, while 5′-NPPOC 2′-*O*-ALE RNA phosphoramidites were prepared by ChemGenes according to published procedures with various synthesis and purification improvements leading to the isolation of high-quality phosphoramidites in gram quantities.[1]

General Microarray Fabrication Procedure

Slide functionalization

Microarrays were synthesized according to procedures described elsewhere, relating to multiple technical improvements that provide the basis for our current array synthesis protocol.[2] In the paired array approach, glass microscope slides (Schott Nexterion Glass D) are silanized using *N*-(3-triethoxysilylpropyl)-4-hydroxybutyramide (Gelest SIT8189.5). The silane reagent (10 g) is diluted into 500 ml EtOH/H_2O 95:5 + 1 ml AcOH and the slides are submerged in the functionalization solution for 4 h at room temperature (r.t.) with gentle shaking. The slides are then washed twice in a 500 ml EtOH/H_2O 95:5 + 1 ml AcOH for 20 min at r.t., transferred into a dry, clean rack and cured overnight in a pre-heated oven at 120 °C under vacuum. After functionalization, the silanized slides are kept in a desiccator until further use. Prior to functionalization, one of the slides is drilled at two positions with a 0.9 mm diamond bit, washed then rinsed in an ultrasonic bath for 30 min.

Nucleic acid synthesis by photolithography

The instrumental setup for the synthesis of microarrays by photolithography consists of four interconnected devices: a DMD, a UV source traversing a series of optical elements, a computer and an automated DNA synthesizer (Expedite 8909, PerSeptive Biosystems). The UV source, a 365 nm high-power UV-LED (Nichia NVSU333A), produces UV light that is first homogenized by passing through a square cross section light-pipe before reflecting on the DMD, where the mirrors are tilted in either an ON or an OFF position. The reflected image off the ON mirrors is then projected onto an Offner relay optical system, providing a 1:1 image of the light pattern on to

the synthesis surface. UV illumination of the slide triggers the removal of the 5′-NPPOC protecting group only at defined locations ("features"), corresponding to the pattern of ON mirrors. The glass slides are encased in a reaction chamber attached to the DNA synthesizer, which controls the delivery of reagents to the surface. The computer synchronizes the exposure to UV with the synthesizer and instructs the DMD to tilt its mirrors in the required position. Light exposure is performed at an irradiance of ~100 mW/cm^2 for 60 s in order to reach a radiant energy density of 6 J/cm^2. During UV exposure, the slides are covered with a solution of DMSO containing 1% (w/w) imidazole. Besides the additional communication with the computer, the DNA synthesizer operates in a similar manner to traditional solid-phase synthesis *via* the phosphoramidite chemistry:

- DNA and RNA phosphoramidites are diluted as 30 mM solutions in dry ACN
- Dicyanodiimidazole (DCI) 0.25 M in ACN is used as the activator
- The oxidation step is performed using a mixture of I_2 in pyridine/H_2O/THF

DNA phosphoramidites are protected with a *tert*-butylphenoxyacetyl protecting group (tac) for dA, *i*PrPac for dG and isobutyryl for dC. They are coupled for 15 s, followed by drying of the surface with helium for 10 s, a short (3 s) oxidation step and finally the exposure to UV. In RNA synthesis, coupling time is the only change to the above protocols, with 5 min coupling time for rA, rC and rG, and 2 min for rU.

<u>Synthesis area, number of features and density</u>

The DMD is a digital light processor containing an array of 1024 × 768 mirrors with a ~14 µm pitch (0.7 XGA). Thus, the total number of individually addressable features amounts to 786432, each feature being 14 × 14 µm in size. All 786432 features, or "spots", are contained within a synthesis area of ~1.4 cm^2. The number of features used during microarray photolithography depends on the type of experiment and the "complexity" of the design (number of sequence permutations, control sequences and replicates). Higher densities for the photolithographic synthesis of nucleic acids microarrays may be obtained using higher resolution DMDs, such as those of dimensions 1920 × 1080 or 4096 × 2160 (~2 and ~9 million mirrors, and a ~11 µm and 7.3 µm pitch, respectively). Alternatively, higher spot densities could in principle be reached without the need for higher resolution DMDs *via* optical demagnification.

Deprotection, Hybridization and RNase H assays

DNA microarrays

After synthesis, the slides were deprotected in a 1:1 solution of ethylenediamine (EDA) in ethanol for 2 h at r.t. (50 ml in a staining glass jar), washed in deionized water, dried in a microcentrifuge then stored in a desiccator until further use.

RNA microarrays

After synthesis, the slides were deprotected in a 2:3 solution of anhydrous Et_3N in ACN for 1h30 at r.t. (50 ml in a falcon tube) with gentle agitation. The arrays were then rinsed twice in ACN (20 ml), dried in a microcentrifuge then transferred into a 0.5 M solution of hydrazine hydrate (1.2 ml) in pyridine/AcOH 3:2 (50 ml in a falcon tube) for 2 h at r.t. The arrays were washed twice in ACN (20 ml each), then dried in a microcentrifuge. Finally, for microarrays containing DNA and RNA nucleotides, a final deprotection step consisted in shaking in a 1:1 solution of EDA in EtOH for 1 h at r.t. The resulting deprotected arrays were washed with sterile H_2O (2 × 20 ml), dried then stored in a desiccator until further use.

Hybridization

Deprotected microarrays were hybridized in a self-adhesive hybridization chamber (Grace BioLabs SA200) filled with a 300 µl of a 10 nM solution of Cy3-labelled complementary strand (Eurogentec) in hybridization buffer (0.1 M 2-(*N*-morpholino)ethanesulfonic acid, 0.9 M NaCl, 20 mM ethylenediaminetetraacetic acid, 0.01% Tween20, 0.05% bovine serum albumine (BSA)). The microarrays were covered in aluminum foil and placed in a hybridization oven (Boekel Scientific) at a slow rotation rate for 2 hours. The assay was performed at 42 °C for the 25 and 28mers whose sequences are given below:

- 25mer on array (sequence given in DNA form):
  5′-GTCATCATCATGAACCACCCTGGTC-3′
- 25mer complementary strand (DNA):
  5′-Cy3 GACCAGGGTGGTTCATGATGATGAC-3′
- 28mer on array (full match sequence, RNA form):
  5′-UUACCAUAGAAUCAUGUGCCAUACAUCA-3′
- 28mer complementary strand (DNA):
  5′-Cy3 TGATGTATGGCACATGTATTCTATGGTTTAA-3′

After hybridization, the chamber was stripped off and the array washed sequentially in three wash buffers: first in Non-Stringent Wash Buffer (SSPE; 0.9 M NaCl, 0.06 M phosphate, 6 mM ethylenediaminetetraacetic acid, 0.01% Tween20) for 2 min, then in Stringent Wash Buffer (100 mM 2-(*N*-morpholino)ethanesulfonic acid, 0.1 M NaCl, 0.01% Tween20) for 1 min and in Final Wash Buffer (0.1X sodium saline citrate) for a few seconds. The arrays were then dried by centrifugation and scanned in a microarray scanner at either 2.5 μm or 5 μm resolution (GenePix 4400A or 4100A respectively, Molecular Devices) with an excitation wavelength of 532 nm. The recorded fluorescence intensities are reported as arbitrary units and were extracted from the scanned images using NimbleScan (Roche NimbleGen) and further processed using Excel.

The signal/noise ratios in hybridization experiments on DNA and RNA arrays were found to be largely similar and to vary between 300:1 and 500:1, or in other terms, hybridization intensities between 10000 and 30000 a.u. were recorded with background levels as low as ~45 a.u.

RNase H assay

A microarray containing the 25mer DNA and RNA sequences was first hybridized to the complementary, Cy3-labelled DNA strand as described above. A solution of 5 Units of RNase H (New England Biolabs) in RNase H buffer (75 mM KCl, 50 mM Tris-HCl pH 8.3, 3 mM $MgCl_2$, 10 mM dithiothreitol) was pipetted in a hybridization chamber attached to the array. The assay was performed for 1 h at 37 °C in a hybridization oven, after which the chamber was removed, the array quickly washed in Final Wash Buffer, dried in a microcentrifuge and scanned. Next, the remaining duplexes on the microarray surface were washed off in H_2O at 37 °C for 10 min. The array was dried, scanned, revealing fluorescence values reduced to background levels. Finally, the microarray was rehybridized to the same Cy3-labelled complement at 42 °C for 2 h according to the procedure described above.

Determination of the coupling efficiency

To measure the coupling efficiencies of DNA and RNA phosphoramidites, we employed the method of terminal labelling. Homopolymers of a single base and of various lengths, 1 to 12 nucleotides, were synthesized on multiple microarrays. The DNA and RNA versions of each homopolymer were synthesized in parallel on the same array. *In toto*, four microarrays were fabricated:

- Array #1 containing: poly-dA (1 to 12-nt) and poly-rA (1 to 12-nt)
- Array #2 containing: poly-dC (1 to 11-nt) and poly-rC (1 to 11-nt)
- Array #3 containing: poly-dG (1 to 12-nt) and poly-rG (1 to 12-nt)
- Array #4 containing: poly-dT (1 to 12-nt) and poly-rU (1 to 12-nt)

Each homopolymer of each length is terminally labelled with Cy3. Terminal labelling consists in two consecutive coupling events of Cy3 phosphoramidite (50 mM, Link Technologies) for 5 min each. After each DNA or RNA phosphoramidite coupling, a capping step is performed with the additional coupling of DMTr-dT phosphoramidite (30 mM, 1 min). Indeed, since microarray synthesis by photolithography bypasses the use of an acidic detritylation event, coupling with a DMTr-protected monomer can essentially be regarded as capping. In addition, a certain number of NPPOC-dT couplings are performed on each homopolymer so as to keep a total sequence length of 12-nt (**Figure S1**). For example, the sequence dG_2 was synthesized over a dT_{10} oligonucleotide, and dG_8 over a dT_4. This is important, as the intensity of the Cy3 dye is known to be dependent on the distance between the fluorophore and the surface of the array. However, those preliminary dT couplings are performed without capping. Finally, each homopolymer receives a final uncapped 5′-dT_{10} linker, regardless of oligonucleotide sequence, so as to distance the fluorophore from other nucleobases which are known to influence the intensity of Cy3 fluorescence.[3]

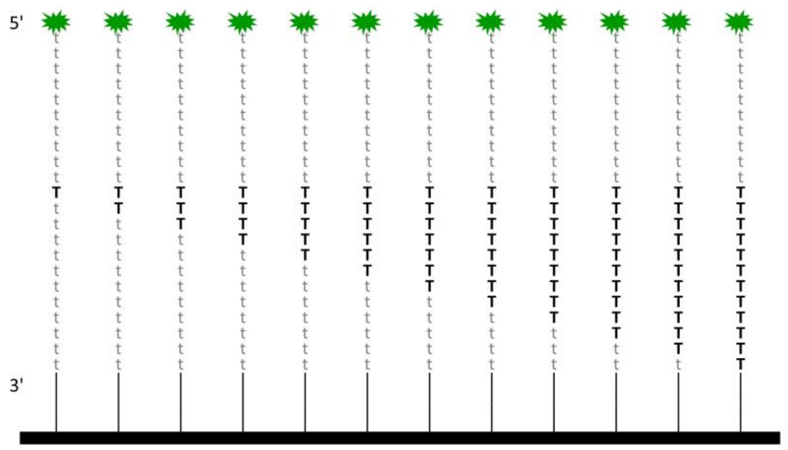

Figure S1. Schematic representation of the sequences synthesized on a microarray for the determination of the coupling efficiency of a given phosphoramidite (here, dT). t = uncapped dT; T = capped dT. Each homopolymer is terminally labelled with Cy3.

After synthesis, the arrays are washed in ACN for 1 h to reduce background fluorescence, dried then scanned and the data extracted with NimbleScan. The decrease in fluorescence as the number of couplings increases follows the mathematical model of an exponential decay. The extracted data, corrected for background and normalized to the most fluorescent sequences (typically dX_1 or rX_1), was further analyzed on SigmaPlot (Systat Software). The plotted curve of normalized fluorescence values was fit to an exponential decay curve $y = ae^{-bx}$ where y is the fluorescence intensity, x the number of couplings, a the maximum of fluorescence intensity and $1 - b$ the stepwise coupling efficiency. The measured coupling efficiencies for DNA phosphoramidites, at a coupling time of 15 seconds each, were:

- dA: 99.9%
- dC: 99%
- dG: 97.7%
- dT: 99.8%

Representative curves, and their fit, for the normalized fluorescence intensities of rU_x and dT_x sequences are shown in ***Figure S2***:

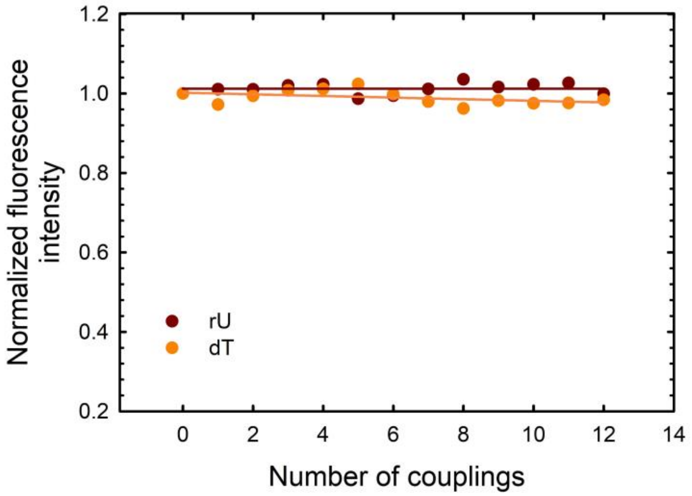

Figure S2. Fluorescence intensity of Cy3-labelled rU_x and dT_x sequences as the number of *x* couplings increases. The plots are fitted to an exponential decay curve from which the stepwise coupling efficiency is obtained. The changes in fluorescence intensity appear minimal due to the very high coupling efficiencies for all phosphoramidites.

<u>Deprotection of Cy3-labelled RNA arrays and RNA degradation</u>

The microarrays used for the determination of coupling efficiency were subjected to the RNA/DNA deprotection protocol, with the intention of monitoring the decrease of fluorescence for deprotected RNA oligonucleotides relative to the corresponding DNA sequences. Arrays were treated first in anhydrous Et_3N/ACN 3:2, 1h30 at r.t., washed in ACN, dried then scanned. Next, the arrays underwent 2′-OH and base deprotection in hydrazine (0.5 M hydrazine hydrate in pyridine/AcOH 3:2, 2 h, r.t.) then EDA (EDA/EtOH 1:1, 1 h, r.t.), washed in ACN, H_2O, dried then scanned. The decrease in fluorescence for RNA oligonucleotides is markedly higher for the longer sequences (rX_{12}) and, for those reasons, only the decrease in fluorescence intensities for rX_{12} constructs are reported. We found a decrease of 30% of the fluorescence intensity relative to the Cy3-labelled DNA after the Et_3N and hydrazine method, and this decrease adds up to 50-60% when performing an extra ethylenediamine step, suggesting indeed degradation (Figure S2). The ability of the deprotected RNA to still strongly hybridize may be the result of a higher duplex stability, but could also potentially stem from variations in oligonucleotide surface density on the feature.[4] RNA degradation should be minimized by shortening the overall deprotection times, and the EDA step

may be avoided altogether with an alternative dG base protection strategy. We also note that oligonucleotides containing a single RNA unit undergo very little degradation.

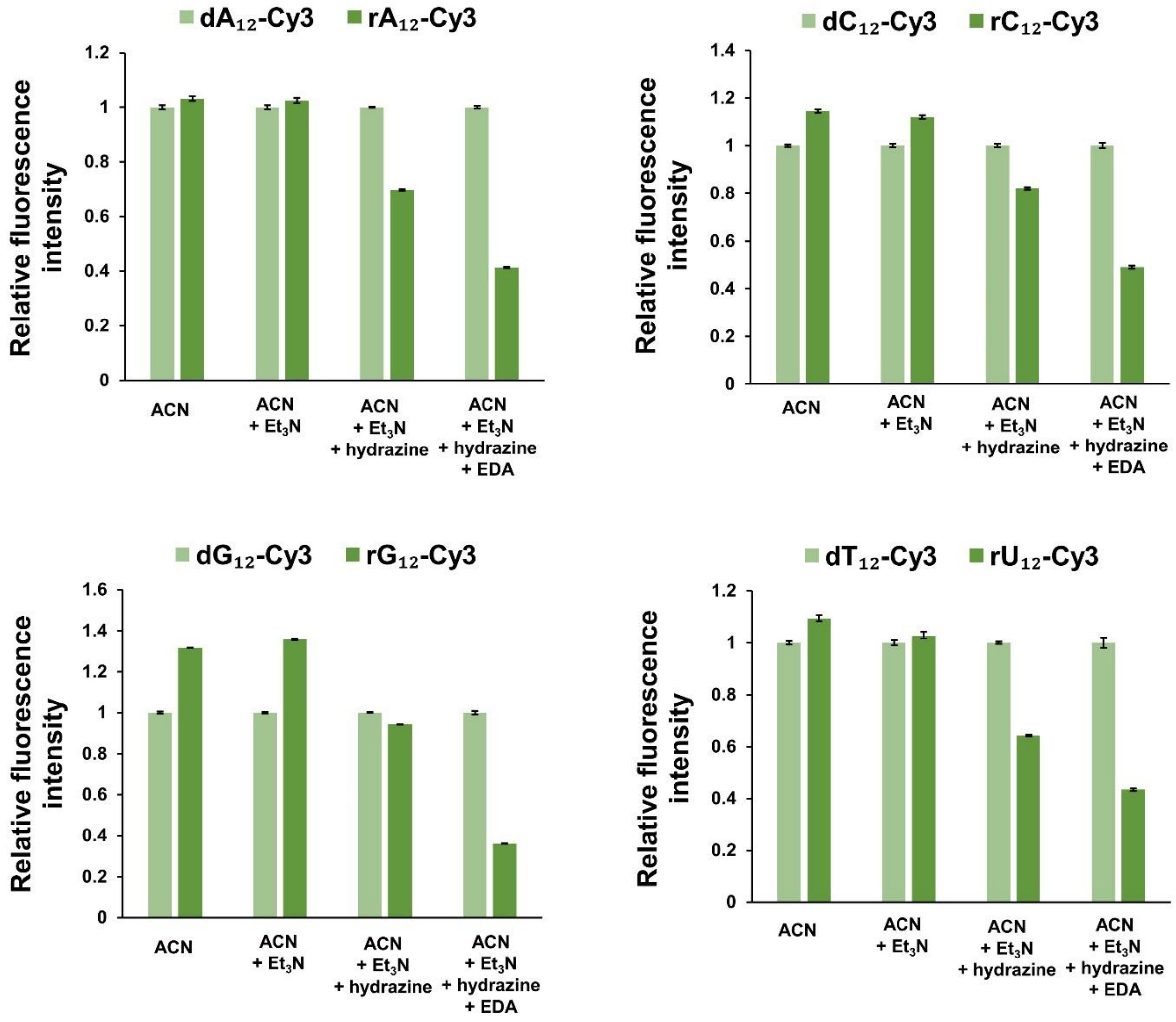

Figure S3. Fluorescence intensities of rX_{12}-Cy3 sequences, relative to those of the corresponding dX_{12}-Cy3, recorded before, during and after RNA deprotection. Error bars are SEM.

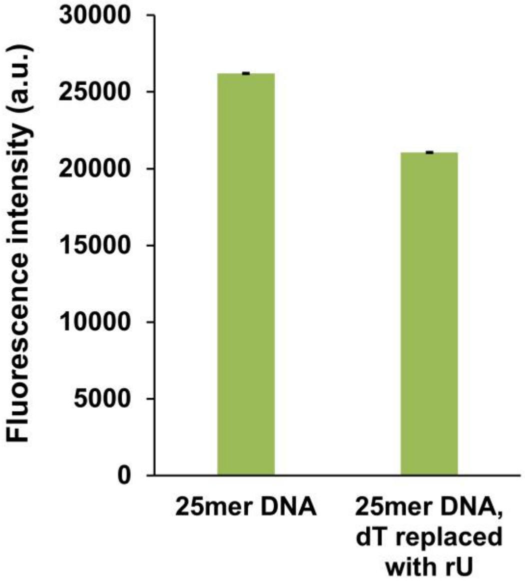

Figure S4. Fluorescence intensities (in arbitrary units) of the 25mer (sequence given above) either in pure DNA form or with all 6 dT positions substituted with rU, hybridized to the same Cy3-labelled complement. Error bars are SEM. The slightly weaker fluorescence signals for the rU-modified 25mer relative to the pure DNA sequence may be attributed to multiple A-B-form helical junctions within the DNA/RNA duplex.

Construction of the 4^9 high-density RNA library

The sequences to be synthesized on the microarray are first written as three separate text files: one for each of the conserved 5′ and 3′ tails, and one for the 9-nt permutation table. The microarray is designed to contain two replicates of each permutation as well as multiple replicates of various single-point mutations of the full-match sequence and multiple replicates of extended and shortened regions. In detail, the RNA library is composed of the sequences listed in Table S1. The sequence text files are uploaded into a custom-built program within MATLAB (MathWorks) that generates a list of masks as well as the order of couplings. The design of the array randomizes the distribution of sequences on the synthesis surface and leaves empty features to serve as background reference. Fiducial features, used for the alignment of the scanned image to the designed layout on NimbleScan, contained the full-match 28mer RNA sequence. An array layout was chosen that uses almost all mirrors for synthesis (~765 000 features) with an unused cross-section in the middle of the layout. A dT_{10} linker is synthesized all on features.

A DNA version of the permuted 4^9 library was also synthesized independently.

After synthesis, the DNA and RNA arrays were deprotected according to the protocols described in the section "Deprotection, Hybridization and RNase H assays". Hybridization was performed with a Cy3-labelled 28mer complementary DNA strand at 42 °C (sequence 5′-Cy3 TGATGTATGGCACATGTATTCTATGGTTTAA-3′).

Table S1. List of RNA sequences synthesized for the high-density RNA library. In bold is the binding region to the FBF-2 protein, ***N*** letters refer to randomized, permuted RNA nucleotides.

Sequence name	*Sequence (5' to 3')* ***N*** *= A, C, G, U*	*Number of features*
Permutation library	UUACCAUAGAAUCA***NNNNNNNNN***CAUCA	524288
Full-match	UUACCAUAGAAUCA**UGUGCCAUA**CAUCA	7000
ACA mutant	UUACCAUAGAAUCA**ACAGCCAUA**CAUCA	7000
U8 to A	UUACCAUAGAAUCA**UGUGCCAAA**CAUCA	2000
U8 to C	UUACCAUAGAAUCA**UGUGCCACA**CAUCA	2000
U8 to G	UUACCAUAGAAUCA**UGUGCCAGA**CAUCA	2000
A7 to U	UUACCAUAGAAUCA**UGUGCCUUA**CAUCA	2000
A7 to C	UUACCAUAGAAUCA**UGUGCCCUA**CAUCA	2000
A7 to G	UUACCAUAGAAUCA**UGUGCCGUA**CAUCA	2000
U3 to A	UUACCAUAGAAUCA**UGAGCCAUA**CAUCA	2000
U3 to C	UUACCAUAGAAUCA**UGCGCCAUA**CAUCA	2000
U3 to G	UUACCAUAGAAUCA**UGGGCCAUA**CAUCA	2000
A9 to U	UUACCAUAGAAUCA**UGUGCCAUU**CAUCA	2000
A9 to C	UUACCAUAGAAUCA**UGUGCCAUC**CAUCA	2000
A9 to G	UUACCAUAGAAUCA**UGUGCCAUG**CAUCA	2000
UGU fixed	UUACCAUAGAAUCA**UGU*NNNNNN***CAUCA	24576
UGU random	UUACCAUAGAAUCA***NNN*GCCAUA**CAUCA	192
NN *random*	UUACCAUAGAAUCA**UGU*NN*AUA**CAUCA	96
NNN *random*	UUACCAUAGAAUCA**UGU*NNN*AUA**CAUCA	384
NNNN *random*	UUACCAUAGAAUCA**UGU*NNNN*AUA**CAUCA	1536
NNNNN *random*	UUACCAUAGAAUCA**UGU*NNNNN*AUA**CAUCA	6144
background		15000

Table S2. Selected fluorescence intensities (in arbitrary units) of the high-density *DNA* and **RNA** libraries of the permuted 28mer sequences hybridized to the Cy3-labelled complementary strand.

		Fluorescence (a.u.)
DNA	background	117
	full-match *TGTGCCATA*	7795
	TGTGCCAAATA	8225
	GCCAUACAT	8730
	TGTGCCCCATA	9060
**RNA**	background	80
	full-match **UGUGCCAUA**	4300
	**GUGCCACAU**	4327
	**GAUGCCAUA**	4530
	**UUUGCCAUA**	4700
	**GCCAUACAU**	4880

Table S3. Number of DNA and RNA sequences with hybridization signals contained within the lowest to highest quartiles of recorded fluorescence signals. Background values correspond to 0 and the highest fluorescence values to 1. Total number of sequences: 263432.

	Number of sequences	
	DNA	**RNA**
1st quartile (0-0.25)	255193	255920
2nd quartile (0.25-0.5)	7801	7263
3rd quartile (0.5-0.75)	431	243
4th quartile (0.75-1)	7	6

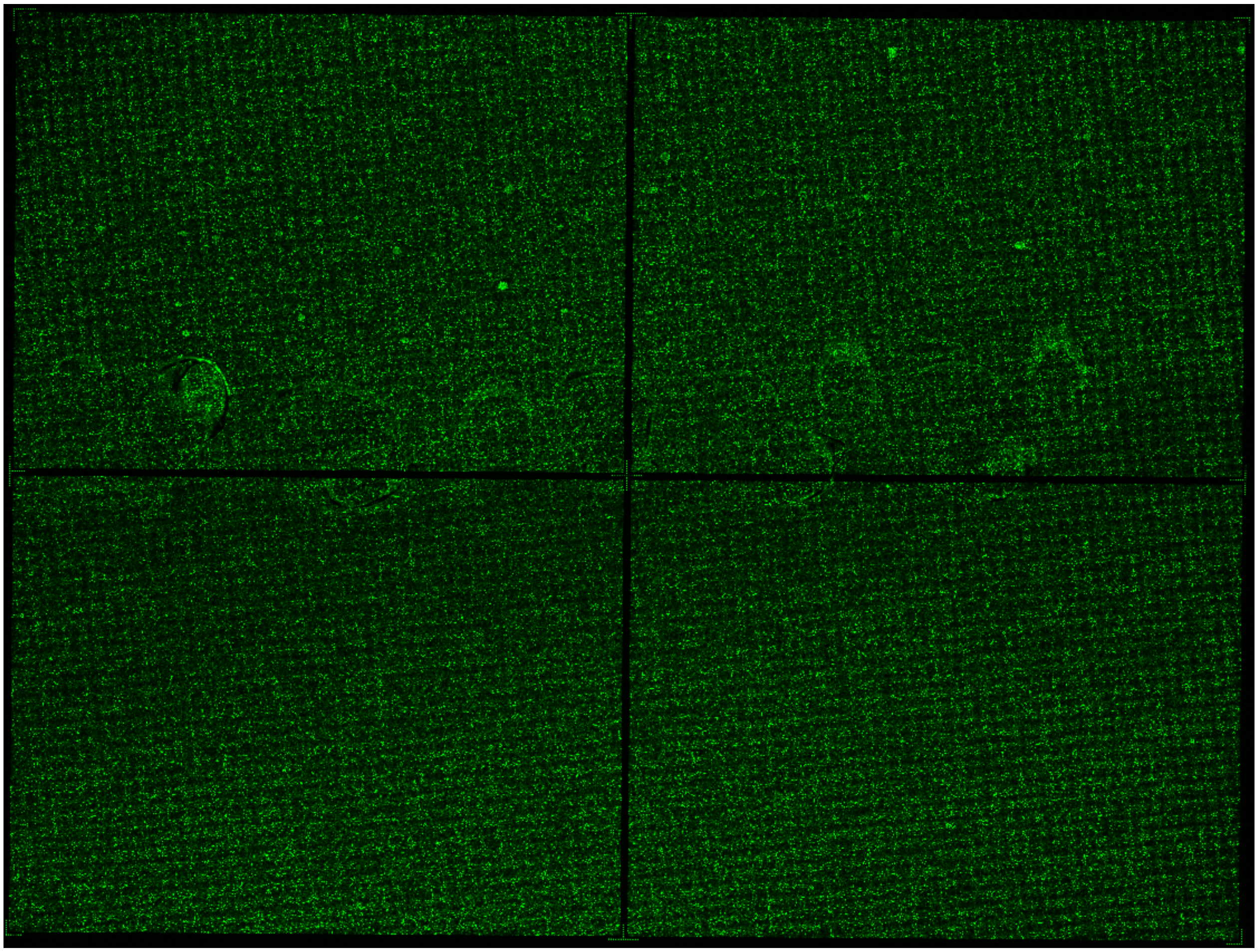

Figure S5. Scanned image of the entire synthesis area of the hybridized high-density RNA microarray (263432 different sequences). Scan resolution: 2.5 µm. Excitation wavelength: 532 nm. PMT gain: 350.

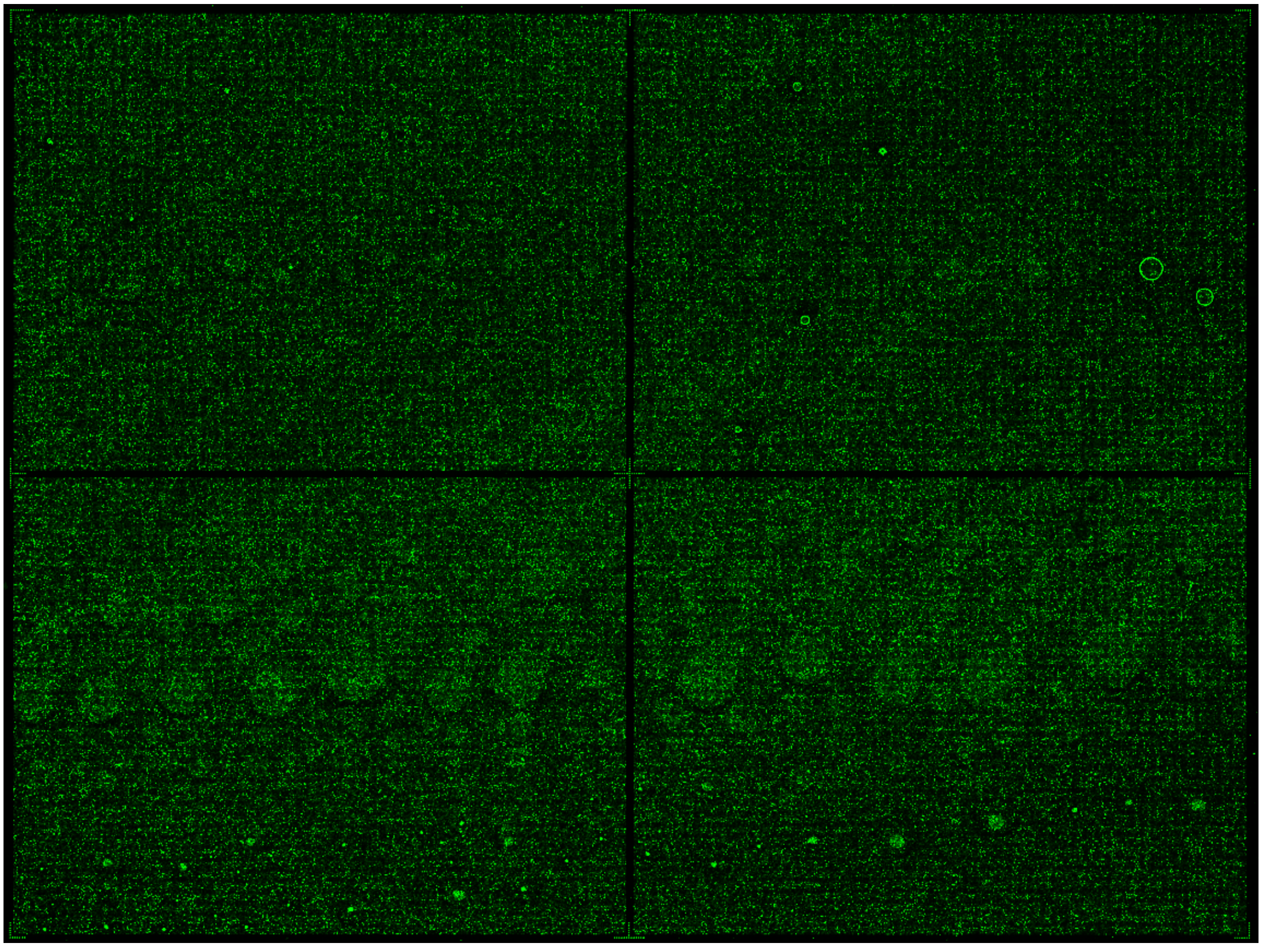

Figure S6. Scanned image of the entire synthesis area of the high-density DNA (263432 different sequences). Scan resolution: 2.5 μm. Excitation wavelength: 532 nm. PMT gain: 350.

RNase HII assays

In a text file are stored all sequences to be synthesized on a microarray, which is then transformed into a series of virtual masks using a custom-built program on MATLAB. The array design was chosen so as to include >30 replicates of each sequence, as well as negative controls and background features in a 4:9 feature size, totaling 85000 features. The synthesis area was covered with 80% background features, and 20% of actual hairpin sequences. Negative controls of the hairpin sequences included a DNA-only sequence as well as a hairpin where the single RNA insert (rU) was introduced in the loop (TCCT) instead of the stem. The sequences were:

* 5′-CCTTATTCCTCCTGGAATAAGG (DNA hairpin)
* 5′-CCTTATTCC*rU*CCTGGAATAAGG (hairpin rU-loop)

The list of all possible sequences in the 5-nt long variable region (shown in red in the DNA-only hairpin sequence) was created using Excel and a built-in "Mix and Match" macro. The complementary part of the variable region was designed on Excel to always be complementary to the varied region. The synthesis of the corresponding array library was performed according to the protocols described above, with a final coupling of Cy3 phosphoramidite at the 5′-end of the hairpin sequences (see "determination of the coupling efficiency"). The linker between the glass slide and the hairpin sequence is a dT_{20}. A capping step was included after each DNA and RNA coupling. In so doing, labelling the 5′ end with Cy3 only becomes possible on the full-length sequence and not on shortmers. This capping step removes a possible bias in data interpretation by attributing total Cy3 fluorescence to the full-length oligonucleotide only, and not to shorter sequences resulting from resumed synthesis after a failed coupling (if any). After synthesis, the arrays were washed in ACN for 1h at r.t., then deprotected according to the protocols described above. Then, a hybridization chamber containing 300 µl of buffer (10 mM KCl; 20 mM Tris-HCl; 10 mM $(NH_4)_2SO_4$; 2 mM $MgSO_4$; 0,1% Triton X-100 pH 8.8; New England Biolabs) was placed over the synthesis area and the array was heated up to 65 °C (5 min) in a hybridization oven then slowly cooled down to r.t. (over 1h). RNase HII (5 µl @ 5 U/µl; New England Biolabs) was then added to the buffer in the hybridization chamber and left to react with the hairpin array library for 1h at 37 °C, after which the chamber was removed, the array washed briefly with sterile H_2O then 0.1X sodium saline citrate, dried and scanned in a microarray scanner at 5 µm resolution. Data extraction and analysis was performed as described before. To calculate the cleavage efficiency, the loss of

fluorescence of a given hairpin between array deprotection and cleavage with RNase HII was normalized to that of the DNA-only hairpin. Background values were subtracted from measured values. The calculation of the cleavage efficiency followed the equations below:

$$A = \frac{I_{hairpin\ before\ RNase} - I_{background\ before\ RNase}}{I_{DNA\ before\ RNase} - I_{background\ before\ RNase}} \qquad (1)$$

$$B = \frac{I_{hairpin\ after\ RNase} - I_{background\ after\ RNase}}{I_{DNA\ after\ RNase} - I_{background\ after\ RNase}} \qquad (2)$$

$$Cleavage\ efficiency = \left(1 - \frac{B}{A}\right) \qquad (3)$$

Where *I* stands for fluorescence intensity (in arbitrary units). Sequence motifs were generated by feeding in a given list of sequences, containing the variable region only, to the WebLogo generator (weblogo.threeplusone.com).[5] After logo generation, the middle nucleotide (A, C, G, or T) was manually replaced with rA, rC, rG or rU, respectively.

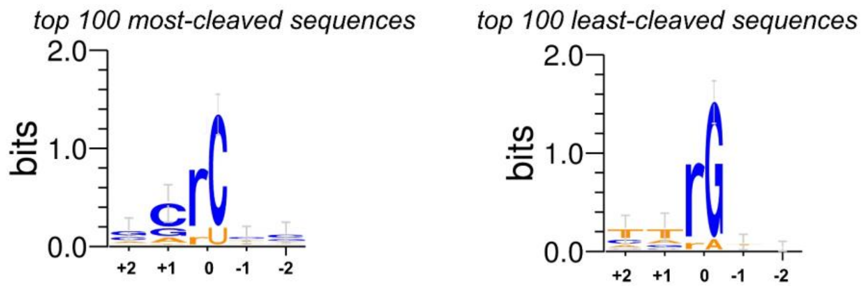

Figure S7. Sequence logos obtained from the list of the top 100 most-cleaved and top 100 least-cleaved sequences amongst all 1024 possible hairpin sequences. Motifs are written in the 5′-3′ direction. The RNA nucleotide is represented as “rX”.

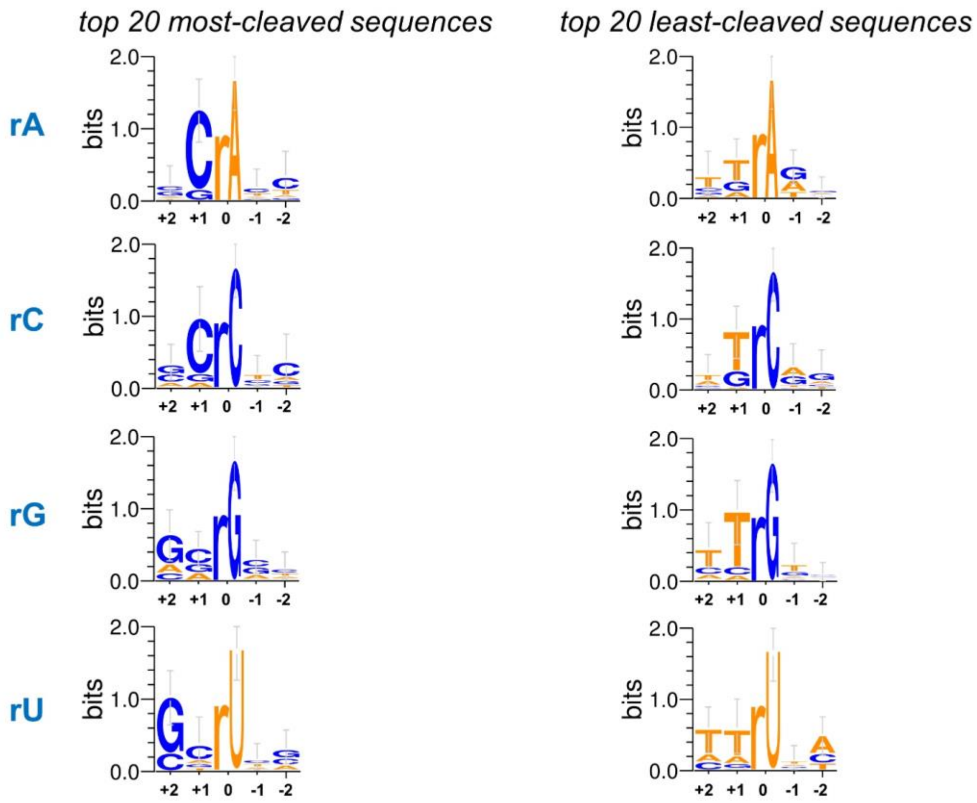

Figure S8. Sequence logos generated by feeding in the list of the top 20 most-cleaved and top 20 least-cleaved sequences for all possible, fixed rX nucleotides into the WebLogo generator. Motifs are written in the 5′-3′ direction.

Further discussion on the results of the RNase HII assay

Table S4. Distribution of the number of sequences (from the 4^5 permutation library) according to the extent of their RNase HII-mediated cleavage.

Total number of hairpin sequences	*1024*
# of sequences with cleavage rates between 40-60%	846
# of sequences with cleavage rates >60%	94
# of sequences with cleavage rates <40%	85

- *Identity of the DNA nucleobases around the RNA insert*

As stated before, cytosine appears to be the preferred 5′ DNA base in the best RNase HII substrates, while the less-cleaved substrates very often show thymine 5′ to the RNA. This over-representation of dC in the most-cleaved sequences and dT in the least-cleaved sequences is observed regardless of the RNA base, though less pronounced in the better-cleaved rU-containing hairpins and the poorly-cleaved rA-containing hairpins (Figure S8). The table below sums up the information found in Figure S7 about the nature of the DNA nucleobase found 5′ to the RNA in the top 20 most-cleaved and top 20 least-cleaved sequences for all possible, fixed rX nucleotides:

Table S5. Nature of the DNA base found 5′ to the RNA nucleotide, when the nature of the RNA base is fixed and when studying the subsets of the most-cleaved and the least-cleaved sequences for each fixed RNA base.

RNA nucleobase	*Cleavage efficiency*	*DNA base 5′ to the RNA*
rA	High	dC
	Low	dT (less pronounced)
rC	High	dC
	Low	dT
rG	High	dC (less pronounced)
	Low	dT
rU	High	dC (less pronounced)
	Low	dT

On the other hand, the sequence motifs from either poor or better hairpin substrates show a less distinct DNA base preference at positions +2, -1 or -2.

- *Number of GC versus AT base pairs in the hairpin stem*

We examined whether abundance of GC base pairs in the stem of the hairpin positively correlates with higher cleavage efficiency, since the corresponding hairpins are expected to be more thermally stable. The melting temperatures for all hairpin sequence combinations were predicted to range between 61 and 86 °C, sufficiently higher than the assay temperature (37 °C) to assume near-complete hairpin formation for all combinations.

We nonetheless found that the 100 most-cleaved candidates had an average of 3.5 GC base pairs (out of 5), hinting at the possibility of higher RNase HII activity on GC-rich constructs. But this observation may be partially explained by the fact that the best substrates for RNase HII activity preferentially show rC as the RNA base, and dC 5′ to the RNA. Whether an increased cleavage rate with a cytosine base at these positions is due to the nature of the nucleobase itself or to the presence of a GC base pair is unclear at this point. However, it seems fair to assume that if the nature of the base pair is central to the cleavage efficiency, then dG and rG nucleotides would have been equally represented within the most-cleaved sequences.

On the other hand, AT-rich hairpins do not necessarily lead to lower cleavage efficiencies, as the 100 least-cleaved hairpins only had an average of 2.5 AT base pairs, and this is in spite of the fact that a dT base is preferentially found 5′ to the RNA in the worst RNase HII substrates.

References

[1] J. G. Lackey, D. Mitra, M. M. Somoza, F. Cerrina, M. J. Damha, *J. Am. Chem. Soc.* **2009**, *131*, 8496-8502.

[2] a) C. Agbavwe, C. Kim, D. Hong, K. Heinrich, T. Wang, M. M. Somoza, *J. Nanobiotechnol.* **2011**, *9*; b) M. Sack, N. Kretschy, B. Rohm, V. Somoza, M. M. Somoza, *Anal. Chem.* **2013**, *85*, 8513-8517; c) N. Kretschy, A. K. Holik, V. Somoza, K. P. Stengele, M. M. Somoza, *Angew. Chem. Int. Ed.* **2015**, *54*, 8555-8559; d) M. Sack, K. Holz, A. K. Holik, N. Kretschy, V. Somoza, K. P. Stengele, M. M. Somoza, *J. Nanobiotechnol.* **2016**, *14*; e) K. Holz, J. Lietard, M. M. Somoza, *ACS Sustain. Chem. Eng.* **2017**, *5*, 828-834; f) J. Lietard, H. Abou Assi, I. Gomez-Pinto, C. Gonzalez, M. M. Somoza, M. J. Damha, *Nucleic Acids Res.* **2017**, *45*, 1619-1632.

[3] C. Agbavwe, M. M. Somoza, *PLoS ONE* **2011**, *6*, e22177.

[4] N. L. W. Franssen-van Hal, P. van der Putte, K. Hellmuth, S. Matysiak, N. Kretschy, M. M. Somoza, *Anal. Chem.* **2013**, *85*, 5950-5957.

[5] T. D. Schneider, R. M. Stephens, *Nucleic Acids Res.* **1990**, *18*, 6097-6100.
